# Longitudinal Study on Sustained Attention to Response Task (SART): Clustering Approach for Mobility and Cognitive Decline

**DOI:** 10.3390/geriatrics7030051

**Published:** 2022-04-22

**Authors:** Rossella Rizzo, Silvin P. Knight, James R. C. Davis, Louise Newman, Eoin Duggan, Rose Anne Kenny, Roman Romero-Ortuno

**Affiliations:** 1 The Irish Longitudinal Study on Ageing, Trinity College Dublin, D02 R590 Dublin, Ireland; silvin.knight@tcd.ie (S.P.K.); davisj5@tcd.ie (J.R.C.D.); louise.newman@tcd.ie (L.N.); dugganeo@tcd.ie (E.D.); rkenny@tcd.ie (R.A.K.); romeroor@tcd.ie (R.R.-O.); 2 Discipline of Medical Gerontology, School of Medicine, Trinity College Dublin, D02 PN40 Dublin, Ireland; 3 Department of Engineering, University of Palermo, 90128 Palermo, Italy; 4 Mercer’s Institute for Successful Ageing, St James’s Hospital, D08 NHY1 Dublin, Ireland; 5 Global Brain Health Institute, Trinity College Dublin, D02 PN40 Dublin, Ireland

**Keywords:** sustained attention to response task, SART, multimodal visualization, threshold, fuzzy clusters, cognition, repeated measures, mobility decline, specificity

## Abstract

The Sustained Attention to Response Task (SART) is a computer-based go/no-go task to measure neurocognitive function in older adults. However, simplified average features of this complex dataset lead to loss of primary information and fail to express associations between test performance and clinically meaningful outcomes. Here, we combine a novel method to visualise individual trial (raw) information obtained from the SART test in a large population-based study of ageing in Ireland and an automatic clustering technique. We employed a thresholding method, based on the individual trial number of mistakes, to identify poorer SART performances and a fuzzy clusters algorithm to partition the dataset into 3 subgroups, based on the evolution of SART performance after 4 years. Raw SART data were available for 3468 participants aged 50 years and over at baseline. The previously reported SART visualisation-derived feature ‘bad performance’, indicating the number of SART trials with at least 4 mistakes, and its evolution over time, combined with the fuzzy c-mean (FCM) algorithm, individuated 3 clusters corresponding to 3 degrees of physiological dysregulation. The biggest cluster (94% of the cohort) was constituted by healthy participants, a smaller cluster (5% of the cohort) by participants who showed improvement in cognitive and psychological status, and the smallest cluster (1% of the cohort) by participants whose mobility and cognitive functions dramatically declined after 4 years. We were able to identify in a cohort of relatively high-functioning community-dwelling adults a very small group of participants who showed clinically significant decline. The selected smallest subset manifested not only mobility deterioration, but also cognitive decline, the latter being usually hard to detect in population-based studies. The employed techniques could identify at-risk participants with more specificity than current methods, and help clinicians better identify and manage the small proportion of community-dwelling older adults who are at significant risk of functional decline and loss of independence.

## 1. Introduction

An increasing number of neurocognitive tests are computer-based. They are used in clinical practice and research to detect neurocognitive dysfunction and/or disorders in adults [1,2]. Very often, the first step in the analysis of such data is the pre-processing and simplification of the raw outputs from computer-based tests, which can lead to loss of relevant information and/or misinterpretation of the results [3,4]. These challenges are even more pronounced in the case of repeated-measures neurocognitive data in large-scale studies.

Commonly employed techniques tend to simplify the raw computer outputs into average features, offering surrogates of overall performance and variability that are easier to process in analyses [5]. However, the loss of primary information could lead to a failure in identifying associations between test performance and clinically meaningful outcomes. Recent works [6] have demonstrated how, especially in Go/NoGo tasks, intra-individual variability (IIV) with its time-dependent feature is an important biomarker in cognitive aging. High IIV has been shown to be strongly correlated with inefficient sustained cognitive processes. Moreover, recent studies [4,7] have demonstrated that the use of the entire raw dataset could help clinicians find important features and peculiar associations that are otherwise hidden in derived measures.

The Sustained Attention to Response Task (SART) is a standard computer-based cognitive test to measure sustained attention, a fundamental executive function for completing tasks that require supervision over time [8]. Sustained attention is a result of the interaction between two different subsystems: vigilance and arousal (alertness) [9,10]. Vigilance allows detection of subtle changes in the environment occurring over long periods of time [9,11], and is related to the activation of a network of cortical areas including the cingulate gyrus, prefrontal cortex and inferior parietal lobule [12,13]. A consistent adequate level of arousal is necessary to detect target stimuli [9]. Electrophysiology and functional neuroimaging studies have demonstrated that arousal is activated through a subcortical network including the thalamus and noradrenergic brainstem structures [14,15]. The SART is a continuous performance reaction-time (RT) task designed to measure attention lapses; participants are required to monitor visual displays acknowledging responses to frequent neutral signals (GO trials), but withholding response when detecting rare targets (NO–GO trials) [5,16]. Commission errors (responding to NO–GO trials) or omission errors (failure to respond to GO trials) reflect lack of vigilance, while the RT is a measure of alertness. Recent findings on analysis of inter-trials SART performances have demonstrated that increased IIV in RT significantly predicted decreased executive control and resistance to distractor in inhibition processes, implying a failure in inhibition performance and an increase in commission errors [6]. Moreover, coherent response patterns of RT from one trial to the next, which seemed to emerge in participants whose RTs varied more widely around the mean, were found to significantly predict a better inhibition performance [6]. Further, a GO-NoGO functional MRI (fMRI) paradigm has been used to study the relationship between metacognitive-executive functions and action-monitoring and response-inhibition [17,18]. During response inhibition, response selection, and target detection tasks the activity of the anterior cingulate cortex (ACC) was heightened. This is particularly important in movement disorders, in which the functioning of the orbito-frontal cortex, responsible for impulse control and decision making, and of the ACC is gravely impacted [17,18]. Besides, recent works have employed SART for the evaluation of loss of insight in frontotemporal dementia (FTD), cortico-basal degeneration (CBG) and progressive supra-nuclear palsy (PSP) patients [19]. Specifically, FTD patients, having more severe damage to the prefrontal structures required for emergent awareness, were particularly impaired in online monitoring of errors compared to the other two patient groups [19]. In older adults, SART has been shown to be correlated with frailty [20], a dysregulation in multiple systems, an emerging geriatric syndrome which results in a state of vulnerability after a stressor event and is manifested as a decline in several organ systems [21,22], and falls efficacy [23]. However, due to its complex granular intrinsic structure, the optimal way to approach the analysis of SART data remains the subject of debate. 

Our previous companion study [7] proposed a novel method to visualise the full information obtained from the SART tests performed by a large sample of older participants in a large population-based study, and a new variable, ‘bad performances’, based on a thresholding method, which could allow detection of a subset of participants considered to have a poor SART performance, and important clinical implications such as future falls and mobility decline [7]. Moreover, recent studies have underscored the importance of the longitudinal investigation of cognitive data in order to individuate possible neurodegenerative disorders as soon as possible [24], as well as functional decline in other physiological systems [25]. However, the longitudinal evolution of SART performance is still poorly understood, as well as the identification of the factors that may play a role in the prediction of functional decline.

Furthermore, recent studies [26,27] have shown the presence of an integrated network of interactions and feedback mechanisms among different physiological systems, particularly between the brain and the loco-motor system. Specifically, correlations have been found between RT and mobility decline [7,25,28], and between poor mobility performance and cognitive decline [25]. Evidence of the interconnections between the cognitive and the loco-motor systems has generated great interest among researchers, and finding a clinical and biological interpretation of the complex networked interactions remains a challenge in medical research.

The most commonly used tests to assess mobility are gait speed measurements, taken at normal pace and during a dual-task (where the participant is required to complete a cognitive task while walking) [29,30], and the Timed Up-and-Go (TUG) test, a well-established test to measure mobility and predict risk of falls in older adults [29,31]. Recent findings have suggested that baseline quantitative gait parameters are significant predictors of cognitive decline and dementia in older adults [25,32,33]. On the other hand, recent studies have demonstrated that older participants with poorer SART performances [7] and poorer choice reaction times [28] may present an accelerated mobility decline and have a higher risk of incident falls [34].

In order to assess global cognitive status, frequently used tests are the Mini-Mental State Examination (MMSE) score as a standard measure of overall cognitive status [35], and the Montreal Cognitive Assessment (MoCA) [36], a more challenging cognitive test compared to the MMSE, which includes executive function, higher-level language, and complex visuospatial processing, and is designed to detect milder impairments. In our previous investigation, we did not find a significant association between baseline SART performance and cognitive decline at 4 years as assessed by MMSE score [7], although SART performance and MMSE score offer two different measures of the same physiological system. Therefore, it was necessary to employ a different technique, which could allow to a higher specificity and detection of a small number of participants with signs of both physical and cognitive decline.

Clustering is an unsupervised machine learning technique that partitions a set of elements into subsets, or clusters, based on similarities among the individual data items [37,38]. Clustering methods are becoming increasingly important in analysing heterogeneity of treatment effects, health conditions and biological features, especially in longitudinal studies [39]. Clustering techniques are mainly divided in two subgroups: ‘hard clustering’ (e.g., K-means algorithm) and ‘soft clustering’ (e.g., Fuzzy C-mean (FCM) algorithm). In hard clustering, each element belongs to one cluster only. Instead, in fuzzy clustering, the clusters can be overlapping, since the probability belonging to each cluster is assigned to each element (a belongingness parameter ranges from 0 to 1) [37,38]. Previous work showed the utility of K-means clustering of cardiovascular data for the discovery of a novel morphological classification of orthostatic hypotension [40,41]. However, hard algorithms are not suitable in most situations for the analysis of biomedical data, since some individuals may or may not be diagnosed with a certain disorder, depending on different conditions [42]. Therefore, fuzzy clustering with its probabilistic approach, could help clinicians to individuate subjects whose classification for certain disorders is not clearly based on classic parameters, but whose trajectory of physiological dysregulation (stability, worsening, improvement) could help to better understand the risk factors and evolution of various medical conditions, including neurodegenerative disorders [42]. Indeed, the FCM algorithm could facilitate ‘precision phenotyping’, which is one of the main challenges of current biomedical research [43,44].

In the present study, which we present as a companion to our previously published paper [7], we aimed to identify relatively homogenous clusters of older adults who shared similar patterns and/or degrees of physiological dysregulation according to the longitudinal evolution of their SART ‘bad performances’, conducted at two separate time points 4 years apart. Specifically, our goals were (i) to detect a likely small specific group of subjects who might have clinical significant decline on both physical and cognitive measures, and (ii) to demonstrate that a soft, probabilistic unsupervised machine learning model of ‘physiologic clustering’ can facilitate this aim.

## 2. Materials and Methods

### 2.1. Dataset

#### 2.1.1. Design and Setting

This study was conducted on data from The Irish Longitudinal Study on Ageing (TILDA), an ongoing nationally representative prospective cohort study of community-dwelling adults. The TILDA dataset contains information on the health, economic, and social circumstances of people aged 50 years and over in Ireland. Participants were randomly recruited based on their geographic location. The full design of the study and cohort characteristics have been previously described [45,46]. Wave 1 of the study (baseline) took place between October 2009 and February 2011 and was organised as follows: (i) a comprehensive health assessment conducted at a dedicated health assessment centre (HAC) and (ii) a computer-assisted personal interview (CAPI). Wave 3 of TILDA was conducted between March 2014 and December 2015 (approximately 4 years after wave 1) and comprised the same modes of data collection as described above. Ethical approvals for each wave were granted from the Health Sciences Research Ethics Committee at Trinity College Dublin, Dublin, Ireland, and all participants provided written informed consent. All research was performed in accordance with the Declaration of Helsinki. In this study we considered data from wave 1 and wave 3 of TILDA, and specifically we considered the merged cohort of both waves, constituted by participants who took part in both waves of HAC and CAPI.

#### 2.1.2. SART Protocol

The SART is a computerised continuous performance RT task [8]. It requires participants to respond to a repeating stream of consecutive digits 1 to 9 (GO trials), but withhold response to the digit 3 (NO–GO trials).

In the SART test, each digit appears for 300 milliseconds (ms), with an interval of 800 ms between digits. The cycle of digits 1 to 9 is repeated 23 times, giving a total of 207 trials. The test lasts for approximately 4 min. Participants are required to press a keyboard key as soon as possible (with RT automatically recorded using Presentation, Neurobehavioral Systems, Albany, CA, USA, version 16.5) for each digit presented. In practice, over the course of the test, many participants lose attention and commit mistakes. Two types of mistakes can be detected in the data: commission errors (i.e., responding to NO–GO trials), which reflect lapses of sustained attention; and omission errors (i.e., failure to respond to GO trials), reflecting a break from task engagement, also corresponding to lapsing attention [5]. In this work, we considered SART data from wave 1 and wave 3 of TILDA. TILDA data is unique in that, to our knowledge, no other population-based study has conducted the SART 4 years apart on the same participants.

#### 2.1.3. Mobility Variables

- *TUG:* TUG measures the time (seconds) taken for a participant to stand up, walk 3 m at normal pace along a line on the floor, turn around, walk back to the chair, and sit down [31]. The test is not just a measure of physical ability, but requires an individual to process instructions, plan and execute movements, focus on the task and avoid distractions. This cognitive component makes the test more complex than straight-line walking. Generally, a cut-off of 12 [29,47] or 14 [48,49] seconds (s) is clinically used to discriminate participants with significant mobility impairment and falls risk. The TUG in wave 1 ($TUG_{1}$) and wave 3 ($TUG_{3}$) were utilised in this study. Given our aim to capture risk of early mobility decline in this relatively healthy community-based sample, we chose the more restrictive cut-off of 12 s to define clinically significant mobility impairment in both waves. Specifically, we defined mobility decline (TUG decline) for a given participant when $TUG_{1}$ was less than 12 s ($TUG_{1}<12$) and $TUG_{3}$ was greater than or equal to 12 s ($TUG_{3}\geq 12$).
- Gait speed: gait speed was assessed using a computerised walkway (4.88 m GAITRite (CIR Systems Inc., Franklin, NJ, USA) pressure sensing mat) [24,33]. Participants performed two walks at usual pace and two walks under dual-task conditions (i.e., reciting alternate letters of the alphabet), starting and finishing 2.5 m before and 2.0 m after the walkway. The measured usual gait speed (UGS) and dual-task gait speed (DTGS) were calculated as an average between the two walks under each condition and did not include the acceleration and deceleration phases. Variable cut-offs have been used in the literature to individuate mobility disability (range 30–100 cm/s) [30] and slow usual pace in older adults (range 80–120 cm/s) [50,51,52]. We considered the UGS at wave 1 ($UGS_{1}$) and at wave 3 ($UGS_{3}$), and defined ‘UGS decline’ for a given participant when $UGS_{1}$ was greater or equal than 100 cm/s ($UGS_{1}\geq 100\;\mathrm{cm}/s$) and $UGS_{3}$ slower than 100 cm/s ($UGS_{3}<100\;\mathrm{cm}/s$). Similarly, we defined *DTGS decline* for a given participant when DTGS at wave 1 ($DTGS_{1}$) was greater or equal than 100 cm/s ($DTGS_{1}\geq 100\;\mathrm{cm}/s$) and DTGS at wave 3 ($DTGS_{3}$) slower than 100 cm/s ($DTGS_{3}<100\;\mathrm{cm}/s$).
- Falls: as part of the CAPI, participants were asked whether they had fallen in the year prior to the interview. We recorded the number of recalled falls in wave 1 ($falls_{1}$) and wave 3 ($falls_{3}$), and defined as ‘new fallers’ participants who had at least 1 fall in the year prior to the examination at wave 3 ($falls_{3}>0$) and no falls in the year prior to the examination at wave 1 ($falls_{1}=0$).

#### 2.1.4. Cognitive Variables

- *MMSE*: Global cognitive function was assessed using the MMSE test, giving participants a score from 0 (minimum) to 30 (maximum) [35]. We considered the MMSE score in wave 1 ($MMSE_{1}$) and wave 3 ($MMSE_{3})$ and, in line with previous recommendations [53], defined as clinically meaningful cognitive decline a decrease of at least 2 points between wave 1 and 3 ($MMSE_{1}\;-\;MMSE_{3}\geq 2$).
- *MoCA*: Cognition was also evaluated using the MoCA. As in the MMSE, scores range from 0 (minimum) to 30 (maximum) [36,54]. In line with previous findings [55], we defined as clinically meaningful cognitive decline a decrease of at least 2 points between wave 1 and 3 ($MOCA_{1}\;─\;MOCA_{3}\geq 2$).

#### 2.1.5. Covariates

Several potentially relevant covariates at wave 1 were considered in this work: (a) features extracted from the SART multimodal visualisation [7], in addition to the traditional SART mean and standard deviation (SD) of RTs (across all trials), both measured in milliseconds; (b) socio-demographic variables: age, sex, and education level (categorised as primary/none, secondary or third/higher); (c) variables expressing the psychological status of participants: anxiety, assessed with the anxiety subscale of the Hospital Anxiety and Depression Scale (HADS-A) [56], which ranges in scores from 0 to 21 (higher scores indicating more symptoms of anxiety); depression, assessed with the Centre for Epidemiological Studies Depression (CES-D) scale [57], which ranges in score from 0 to 60 (higher scores indicating worse depressive status); and (d) variables related to the physical status of participants: whether or not they were taking any antihypertensive medications (coded using the Anatomical Therapeutic Chemical Classification (ATC) [58]: antihypertensive medications (ATC C02), diuretics (ATC C03), β-blockers (ATC C07), calcium channel blockers (ATC C08), and renin-angiotensin system agents (ATC C09)), had history of diabetes, self-reported smoking (categorised as never, past, or current) and alcohol consumption habits (the answer to the question “Do you have a drinking problem?” (yes, no, or I don’t know) was recorded), UGS at baseline, and physical activity status based on the International Physical Activity Questionnaire (IPAQ) (short form) scoring protocol (categorised as low, medium, or high) [59].

### 2.2. Multimodal Visualisation

All analyses and graphical representations were created with MATLAB (R2020b, The MathWorks, Inc., Natick, MA, USA).

#### 2.2.1. Entire Sample

Details and mathematical procedure for the multimodal visualisation, a previously reported method to visualise the individual trial information obtained from the SART test together with global parameters, are described elsewhere [7]. Briefly, the main graph is constituted by a cloud plot, where we represented a spot for each trial and participant, in which the position on *y*-axis indicates the average RT in that trial, and size and colour of the spot indicate the number of mistakes within that trial. Participants sorted by age in ascending order are organised horizontally from youngest (left) to oldest (right). Moreover, additional curves indicating the total number of mistakes, MMSE and TUG, in red, blue, and green, respectively, are superimposed over the first graph.

#### 2.2.2. Thresholded Multimodal Visualisation

We defined as ‘bad performance’ a trial where the participant committed at least 4 mistakes out of 9 possible actions [7], and represented in a second graph only the SART “big spots”, corresponding to bad performances. All of the above-mentioned notations regarding the coordinates, size and colour of the spots still apply. Likewise, the curves representing the number of mistakes, MMSE score, and TUG were now limited only to participants who had at least one bad performance. 

#### 2.2.3. Longitudinal Multimodal Visualisation

We undertook the multimodal visualisation for wave 1 and wave 3, considering two different color maps (‘copper’ and ‘parula’ respectively) to code the percentage of mistakes within a given trial for each spot and participant. Similarly, the curves representing the global parameters were shown for wave 1 and for wave 3. We note that for the visualisation for wave 3 the merged cohort for waves 1 and 3 was used.

Furthermore, we represented the thresholded multimodal visualisation, which showed only participants who had at least 1 bad performance, for waves 1 (dark brown / black) and 3 (blue) in the same graph, indicating on the *x*-axis the age of participants of the merged cohort at wave 1, and showing the curves representing the total number of mistakes, MMSE score and TUG both in wave 1 and wave 3. We note that this graph showed only participants who had at least 1 bad performance in wave 1 and/or wave 3. Therefore, for those participants who had bad performances in waves 1 and 3, brown and blue “big spots” were visible in the graph, for those who had bad performances only in wave 1 only brown “big spots” were visible, while for those who had bad performances only in wave 3 only blue “big spots” were visible.

Moreover, the curves indicating the MMSE score and TUG in wave 3 were constituted by (i) a regular line for those participants who had bad performances only in wave 3, and (ii) stars for those participants who had bad performances also, or only, in wave 1.

### 2.3. Fuzzy Clusters

Clustering is an unsupervised machine learning technique that partitions a set of elements into subsets, or clusters, such that:

1. elements of the same group are similar to each other (they are ‘close’ to each other),
2. elements in different groups are dissimilar (they are far apart from each other).

The concept of ‘distance’ can be represented by one or more continuous variables, and each element of the initial set has a value for each variable considered.

Clustering algorithms can be divided into two subgroups: ‘hard clustering’ and ‘soft clustering’. In hard clustering, each element belongs to one and only one cluster. Instead, in soft clustering, or ‘fuzzy clustering’, the probability to belong to each cluster is assigned to each element, therefore the subdivision is not sharp, but ‘fuzzy’ [37,38].

One of the widely used soft clustering algorithms is the Fuzzy C-means clustering (FCM) algorithm [60]. FCM, as all the other clustering algorithms, uses an iterative process to partition the set of elements into subsets [39,61]. A full mathematical explanation of the FCM algorithm is given in Appendix A.

We considered the merged cohort (waves 1 and 3) and applied the FCM algorithm to classify the set of participants into 3 clusters $C_{1},\;C_{2},\;C_{3}$, using a ‘distance’ based on the variable ‘bad performance’ [7] at wave 1 and wave 3. At the end of the partitioning procedure, each participant had 3 probability scores $p(C_{1}),\;p(C_{2}),\;p(C_{3})$ (range [0, 1]), one for each cluster. For each participant, we considered the maximum of the 3 probability scores and assigned the participant to the corresponding cluster.

#### Elbow Method

Clustering algorithms depend on a predetermined number of clusters, whereas, in practice, clusters are usually unpredictable. The ‘elbow’ method is one of the most commonly used methods to individuate the optimal number of clusters in which a set of elements should be partitioned [62]. 

For each number of clusters, we can consider the Within-Clusters-Sum of Squared errors (WSS), which gives the sum of the square distances between each point of a certain cluster and its centroid. It can, then, be considered as a function depending on the variable *c*, the number of clusters.

$$WSS\left(c\right)=\overset{c}{\underset{i=1}{\sum }}\underset{x\;\in \;C_{i}}{\sum }\left({\left(x_{w1}-q_{i_{w1}}\right)}^{2}+{\left(x_{w3}-q_{i_{w3}}\right)}^{2}\right)$$

where $x_{w1}$ and $x_{w3}$ are the value of the variable ‘bad performance’ for the participant *x* belonging to cluster $C_{i}$ at wave 1 and wave 3, respectively. The same is applied to $q_{i_{w1}}$ and $q_{i_{w3}}$, where $q_{i}$ is the centroid of the cluster $C_{i}$ [62,63].

According to the elbow method, the “best” number of clusters *c* corresponds to the first point of the minimum of the function WSS(c), namely the number of clusters for which the function WSS(c) starts to decrease, in other words, the clusters start to be dense, which is the goal of “good” clustering. Generally, the function WSS(c) will decrease eventually, having a large number of clusters, because the more clusters there are, the finer the partition. So, what is important is the point where WSS will start to decrease for an increasing number of clusters, which will have, in the graph, the shape of an ‘elbow’.

We considered WSS(c), c=1,…,10 and chose the optimal number of clusters.

### 2.4. Statistical Analysis

#### 2.4.1. Longitudinal Study on SART

We considered the SART performances of participants in the merged cohort at waves 1 and 3. In particular, we analysed the longitudinal evolution of the variable ‘bad performance’ [7] between the two waves, and produced two types of histogram: the first one showed the individual trial mistakes distribution at wave 1 and wave 3, and the second showed the distribution of the variable bad performances at the two waves. In more detail, the first graph was constituted by two histograms, one for each wave; each histogram had 10 bars, for 0, 1, 2, …, or 9 mistakes within a single trial, respectively. Each bar was made of many thin vertical lines, one for each participant who had committed in at least 1 trial as many mistakes as indicated by the corresponding bar, represented in different colours, consistent across different bars; the height of these thin vertical lines indicated the frequency of that number of mistakes committed, namely the number of trials in which that participant committed the number of mistakes indicated by the bar. For example, the fifth bar contained all the vertical lines corresponding to participants who committed 4 mistakes in at least 1 trial; the height of a given line indicates the number of trials in which the corresponding participant committed 4 mistakes. Therefore, summing up the height of the vertical lines contained in the 4th, 5th, …, 9th bars, we can obtain the number of bad performances in that wave, as per definition of ‘bad performance’ [7]. The second histogram simply showed the number of bad performances for each participant at waves 1 and 3 distinguishing the two waves by colour: blue for wave 1 and red for wave 3. The two histograms were created in MATLAB.

We then statistically compared the distribution of ‘bad performance’ at wave 1 and wave 3 using the Wilcoxon test, a nonparametric test used to compare related samples [47,48]. We also tested the variable bad performances at wave 1 and wave 3 for potential trends, using the Spearman’s rank correlation coefficient [41,64,65].

We dichotomised the variable ‘bad performance’ at wave 3 and assigned 1 to those participants who had at least 1 bad performance at wave 3, and 0 otherwise. Binary logistic regression (BLR) models were used to predict the binary outcome of bad performances at wave 3, considering as potential predictor the continuous variable bad performances at wave 1. Covariates at wave 1 were used in four different regression models to gradually determine the robustness of the predictor: model 1, with just the predictor; model 2, which was model 1 additionally adjusted with mean RT and SD RT; model 3, which was model 2 controlled by age, sex and education level; and model 4, which was the fully adjusted regression model, considering also all the other covariates mentioned in Section 2.1.5 (anxiety, depression, hypertensives, diabetes, smoking, alcohol, UGS baseline and IPAQ). We reported the odds ratio (OR) with corresponding 95% confidence interval (C.I.) and *p*-value for each independent variable in the model. The OR expresses the odds that an outcome will occur in the presence of an independent variable, compared to the odds that the outcome will occur in the absence of that variable; therefore if OR>1 the independent variable influences positively the odds of the outcome, if OR<1 the independent variable influences negatively the odds of the outcome, i.e., it is “protective” against the outcome, and if OR=1 the independent variable does not influence the outcome [30,50]. The same four different BLR models were applied considering the same covariates mentioned before but substituting the ‘number of bad performances’ with the global variable ‘number of total mistakes’ in the whole SART task, and the ‘number of mistakes in good performances’, both variables at wave 1 [7]. Of note, every time we applied the binary logistic regression model (whether adjusted by covariates or not) we considered only one of these three potential predictors, because we were interested to test whether bad performances at wave 1 could be used independently from the other predictors. Each adjusted model, considering the three different predictors separately, had been tested for multi-collinearity (based on Spearman’s correlation). We compared the OR of the three predictors, whilst noting the degree of overlap in the 95% C.I.s and the corresponding *p*-values.

All the aforementioned statistical tests were performed in IBM SPSS Statistics version 27 (IBM Corp., Armonk, NY, USA). Statistical significance was set at p<0.05 throughout.

#### 2.4.2. Clusters Characterisation

We performed a comprehensive characterization of participant clusters at each wave. Particularly, for the main variables (TUG, falls, UGS, DTUGS, MMSE, MOCA) we checked the statistical difference between the distributions of the same variables at the two waves with the Wilcoxon test.

Moreover, we computed the inter-wave change of a variable between wave 1 and wave 3 and compared it between clusters. For each participant in a given cluster, we considered the relative difference of the same variable between waves 1 and 3 and referred to the value at wave 1, e.g., $\left(UGS_{1}-UGS_{3}\right)/UGS_{1}$ for *UGS*. Then, we compared the distributions of these values between different clusters using the Mann-Whitney U test, a non-parametric test used to compare non-related samples [66], and repeated the procedure for the other main variables.

Finally, we measured the proportion of decline within each cluster. Particularly, we computed the percentage of participants who manifested a decline for a given variable, according to the cut-offs defined in Section 2.1.3 and Section 2.1.4, within each cluster. We then applied the $\chi^{2}$- test to check whether the differences in proportions between clusters were statistically significant. In fact, the $\chi^{2}$- test is used to test the independence between two categorical variables [67,68]. In our case, the two categorical variables considered were (i) the classification in clusters and (ii) whether the participant showed a decline in one of the main variables (*TUG*, *falls*, *UGS*, *DTUGS*, *MMSE*, *MOCA*). If p<0.05, then there was significant dependence between the two categorical variables. Moreover, the $\chi^{2}$-value gives a measure of the dependence: consulting a $\chi^{2}$ distribution table [69], we can see the minimum $\chi^{2}$-value accepted in order to have the dependence between categorical variables considered significant, namely for probability values *p* of $\chi^{2}$ such that p<0.05. The minimum $\chi^{2}$-value accepted depends by the number of degrees of freedom *df*, which corresponds to the number of classes minus one. In our case, we had df=2, since our classes were represented by the 3 clusters, and the corresponding minimum $\chi^{2}$-value accepted was 5.99 [67,69].

## 3. Results

Raw SART data were available for 3468 participants (54.2% women; age: 61.0 ± 7.8 years at wave 1) for the merged cohort (wave 1 and wave 3). Table 1 presents descriptive statistics for the variables used in this work at wave 1 and wave 3.

**Figure 1:**
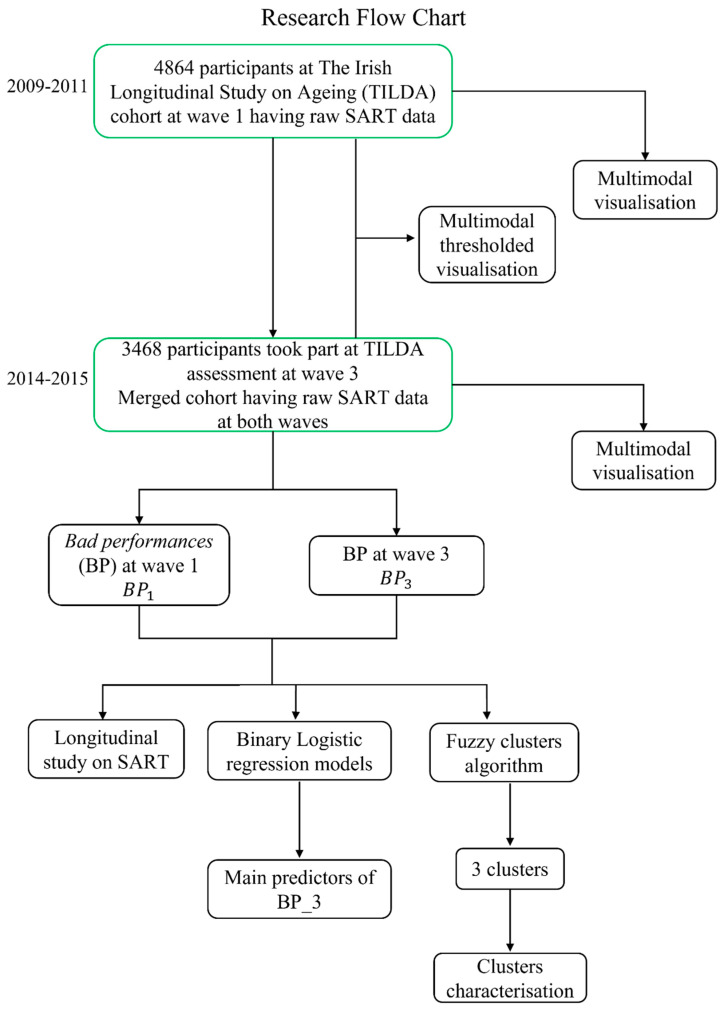
Research flow chart, where the input and output at every phase of analysis are detailed. The flow chart has a color-code: green for data collection, black for output and steps of the analysis.

In Figure 1 we present a flow chart of the present research work, starting from the datasets (cohort at wave 1 and merged cohort at wave 3), reporting on the side the year of corresponding data collection, and indicating with arrows the employed analysis, mentioning the output from certain steps of the analysis, which used as input the dataset or the output of previous steps. Moreover, next to each output of analysis, where appropriate the figures and/or tables are indicated which represent the output obtained. The flow chart has a color-code: green for data collection, black for output and steps of the analysis, blue for figures and red for tables.

### 3.1. Longitudinal Multimodal Visualisation

**Figure 2:**
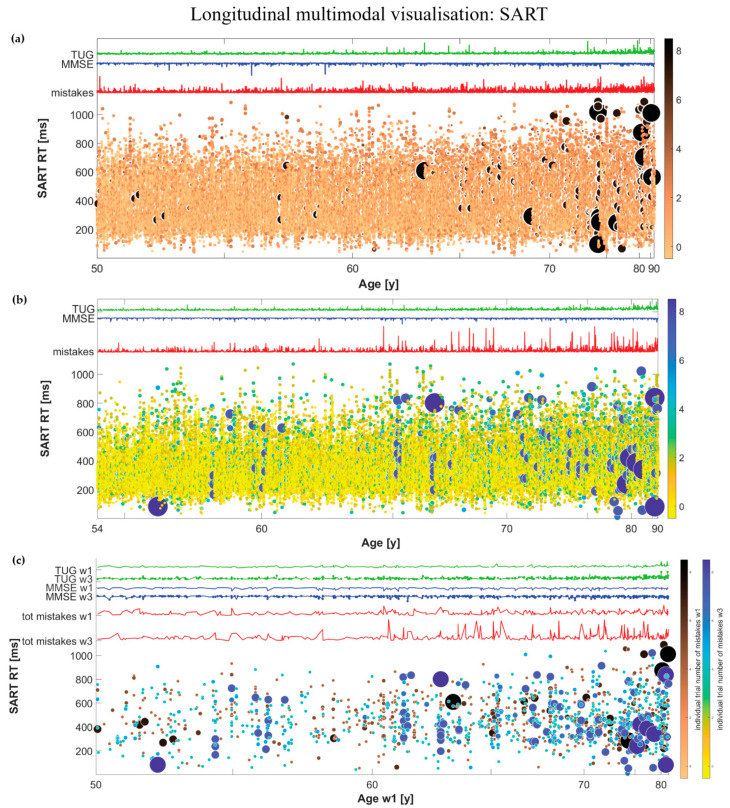
Longitudinal multimodal visualisation of SART data: (**a**) entire cohort at wave 1, (**b**) merged cohort at wave 3, (**c**) only participants with bad performances at wave 1 (dark brown/black big spots) and/or at wave 3 (blue big spots).

Figure 2 shows the multimodal visualisation based on the procedure described in Section 2.2. Figure 2a presents the multimodal visualisation for the entire SART dataset at wave 1 (N=4864 participants) [7]. There were in total 1222 “big spots” representing bad performances for 565 different subjects (11.6% of the sample). Among those aged 50–64, 8.2% had bad performances; among those aged 65–74, 17.9% had bad performances; and among those aged 75 years and older, 33.7% had bad performances. Figure 2b presents the multimodal visualisation for the entire merged cohort at wave 3 (N=3468 participants). There were in total 1244 “big spots” representing bad performances for 403 different subjects (11.6% of the sample). Among those aged 54–64, 6.6% had bad performances; among those aged 65–74, 14.0% had bad performances; and among those aged 75 years and older, 24.1% had bad performances. The density distribution of big spots can be better appreciated in Figure 2c. Figure 2c presents the thresholded multimodal visualisation for the merged cohort (N=3468), only showing data for participants who had at least 1 bad performance at wave 1 (brown/black spots) or only participants who had at least 1 bad performance at wave 3 (dark blue spots). In the merged cohort there were in total 732 “big spots” representing bad performances at wave 1 for 329 different subjects (9.5% of the sample). Among these, only 104 participants also had bad performances at wave 3. Therefore, 225 participants of the merged cohort improved their SART performances, while 299 participants worsened their SART performances.

### 3.2. SART Longitudinal Study

#### 3.2.1. Histograms

**Figure 3:**
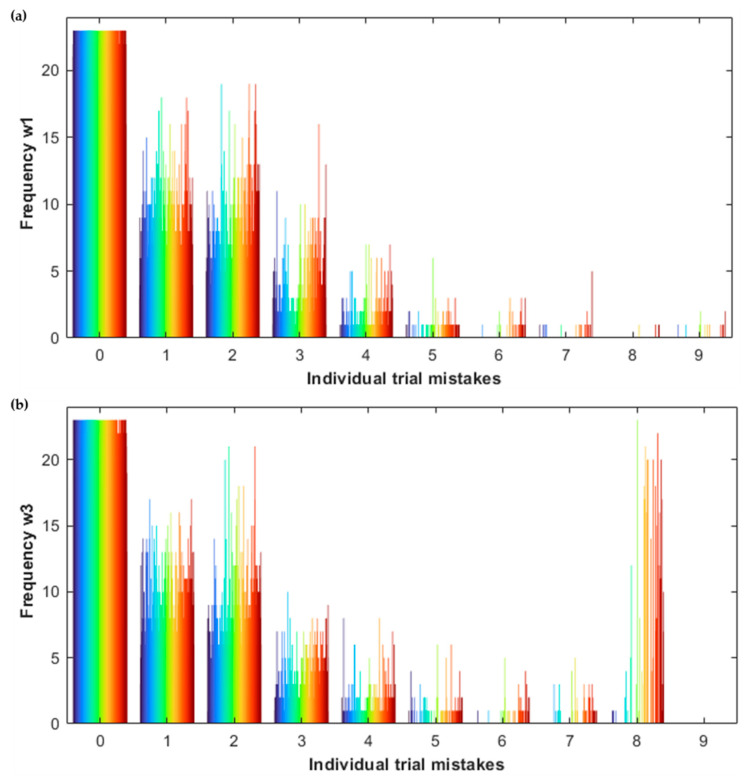
Histograms of distribution of individual trial mistakes at (**a**) wave 1 and (**b**) wave 3. Within each group of bars, the participants are age-sorted from left to right: 50–59 year (blue-light green), 60–69 year (green-orange), 70 year and over (red).

Figure 3 shows the distribution of individual trial mistakes at wave 1 and wave 3, as described in Section 2.4.1, and Table 2 summarises the number of participants in the merged cohort who made 0, 1, 2, … mistakes in an individual trial and how many trials there were in total with the corresponding number of mistakes.

Moreover, Table 2 indicates the change in percentage between wave 1 and wave 3 in number of participants for each individual trial number of mistakes and in total number of trials with the corresponding number of mistakes. Comparing the distributions of individual trial mistakes at wave 1 and wave 3, we note that at wave 3 the number of participants who made 0 mistakes and the total number of trials with 0 mistakes decreased compared to wave 1; the same is valid for 9 mistakes, while for all the other values of individual trial mistakes, the density increased at wave 3, showing a general worsening trend for the SART performances. Besides, the percentage of change between wave 1 and wave 3 increased, with a growing ratio along with the higher number of mistakes in each trial.

**Figure 4:**
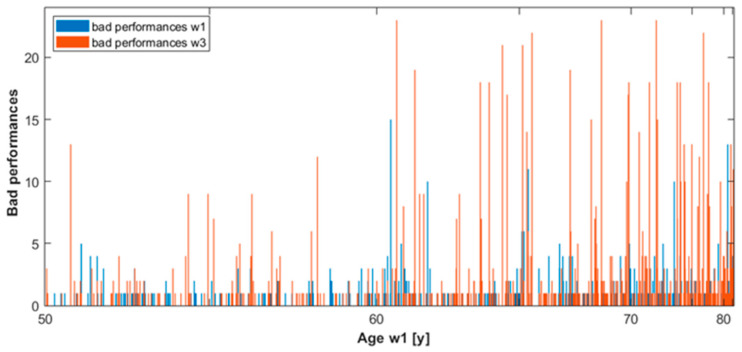
Histograms of the distribution of the variable *bad performances* for each participant at waves 1 (blue) and 3 (red).

Figure 4 shows the distribution of the variable ‘bad performances’ for each participant at waves 1 and 3. We note those blue lines not superimposed by red lines, namely participants with bad performances at wave 1 who did not have bad performances at wave 3.

Moreover, generally the red lines are higher than the blue lines. Therefore, the histogram provides the following information: (i) there were not many consistent bad performance participants between waves, (ii) the number of participants with bad performances increased at wave 3, (iii) the number of bad performances per participant increased at wave 3.

#### 3.2.2. Dynamic Graph

**Figure 5:**
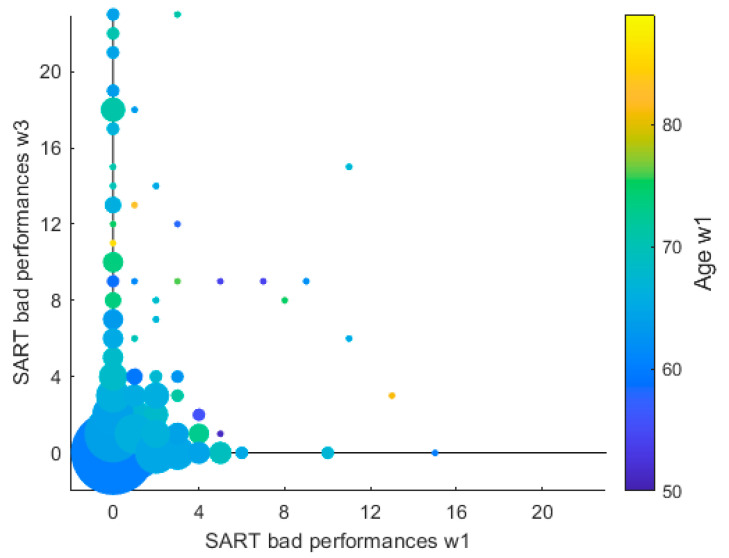
Evolution of the variable bad performances between wave 1 (along the *x*-axis) and wave 3 (along the *y*-axis). The colour of the spots represents the age at wave 1. The size of the spots indicates the density distribution of the variables.

Figure 5 shows the evolution of the variable ‘bad performances’ (BP) from wave 1, on the *x*-axis, to wave 3, on the *y*-axis, where each spot indicates a different value of bad performances (number of bad performances for each participant) present in the distribution of the variable at each wave, i.e., the coordinates of each spot indicates a pair $\left(BP_{1},BP_{3}\right)$, where $BP_{1}$ is the number of bad performances at wave 1, and where $BP_{3}$ is the number of bad performances at wave 3. The size of each spot is proportional to the number of participants that have the same pair $\left(BP_{1},BP_{3}\right)$. We note that 2840 participants (82% of the cohort) had $BP_{1}=0$ and $BP_{3}=0$, while for 23 points in the graph the corresponding pair in $\left(BP_{1},BP_{3}\right)$ had been registered in just one participant. Therefore, using a linear proportion between the size of the spots and the number of participants for the corresponding pair would not make all the spots visible. Thus, we employed a logarithmic transform to the number of participants for each pair $\left(BP_{1},BP_{3}\right)$, and the spots size corresponds to the density of that pair in log scale. Moreover, the color of each spot indicates the age at wave 1 averaged across all participants who registered the corresponding pair of values $\left(BP_{1},BP_{3}\right)$.

The Wilcoxon rank sum test suggested that the distributions of BP at waves 1 and 3 were significantly different from each other: *p* < 0.001. Moreover, no significant trends were individuated between $BP_{1}$ and $BP_{3}$: Spearman’s coefficient was ρ=0.210 at the significance level α=0.01.

### 3.3. Predictive Model for SART Bad Performances

The three potential SART predictors, bad performances, total mistakes and mistakes in good performances, were not normally distributed (Kolmogorov–Smirnov and Shapiro–Wilk normality tests p<0.001, i.e., their distributions were not significantly similar to the normal distribution) [7]. Therefore, we excluded the linear regression model and any other parametric tests and applied binary logistic regression models to predict the presence of bad performances at wave 3 (binary outcome as defined in Section 2.4.1). In every model, the independent variables passed the multi-colinearity test (Spearman’s correlation coefficient |ρ|≤ 0.422 for all pairs at the significance level α=0.01) and satisfied all other logistic regression assumptions [7].

Table 3 shows a comparison of the OR, reporting also the 95% C.I. and *p*-value, for the three predictors in the four different logistic regression models, as defined in Section 2.4.1. In each model, all predictors were significantly associated with the presence of bad performances at wave 3. However, the variable ‘bad performances’ always had a larger OR than that of other predictors, and without overlap of 95% C.I.s, suggesting its larger weight in the prediction of this outcome (*p* < 0.001 in all four models, OR = 1.326, 95% C.I. = (1.167; 1.506) in the fully adjusted model (model 4), i.e., for every one-unit increase in bad performances we would expect an increase of 0.326 in the odds for having bad performances at wave 3.

Table 4 shows the results of the fully adjusted binary logistic regression model 4 where the OR, 95% C.I. for OR and *p*-value for each independent variable in the model are reported. Of note, other significant predictors of the presence of bad performances at wave 3 in model 4 were SD RT, age, and level of anxiety. A third/higher level of education was significantly protective against ‘bad performances w3’, i.e., those who were highly educated were less likely to have a SART bad performance after 4 years. Moreover, comparing the OR of bad performances across different models applied, we noted that it was stronger in model 1, decreased in models 2 and 3, and increased again in model 4.

### 3.4. Fuzzy Clusters

**Figure 6:**
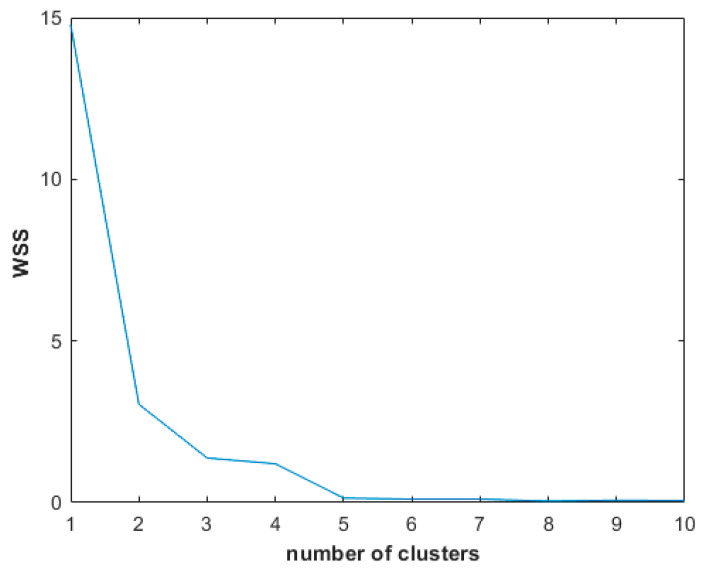
Elbow method applied to the merged cohort (*N* = 3468) showing the Within-Clusters-Sum of Squared errors (WSS) for 1,2,…,10 clusters.

Results of the WSS for 1,2,…,10 clusters of participants in the merged cohort obtained applying the FCM algorithm based on the variable bad performances at wave 1 and wave 3 are shown in Figure 6. According to the elbow method the optimal number of clusters could be three or five. However, applying the FCM for five clusters we obtained an excessively low number of participants in one cluster, which would have not allowed meaningful statistical analysis. Therefore, we opted for three clusters as optimal partition.

**Figure 7:**
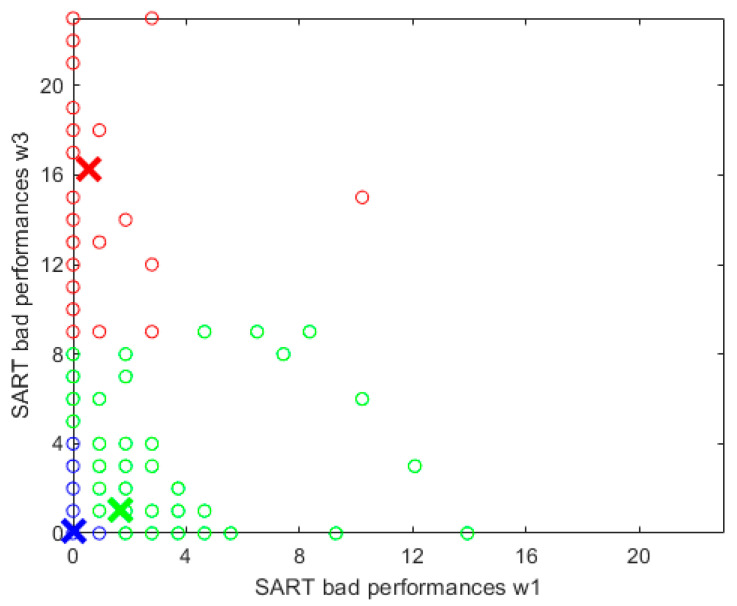
Fuzzy clusters resulted from the FCM algorithm applied to the merged cohort (*N* = 3468) considering the values of bad performances at waves 1 and 3. Each cluster is identified by a color.

Applying the FCM algorithm, we obtained three clusters, presented in blue, green and red respectively in Figure 7, and representing the following types of participants based on their SART performances at waves 1 and 3: the blue cluster comprehended participants who did not have any bad performances at wave 1, or had just one, and maintained a very low number (maximum four) of bad performances at wave 3; the green cluster comprehended participants who had a wide range of values in bad performances at wave 1 (0–15) but did not have more than nine bad performances at wave 3; and the red cluster comprehended participants who did not have more than three bad performances at wave 1 (11 for only one participant) but had bad performances≥9 at wave 3; in some cases even all the 23 SART performances were ‘bad’ at wave 3. The blue cluster was the biggest, containing N=3254 participants, the green cluster had N=177  participants and the red cluster had N=37  participants.

#### 3.4.1. Cluster Characterisation

Table 5 presents descriptive statistics for the variables used in this study at wave 1 and wave 3 for each cluster. Of note, (i) the red cluster was the oldest on average, (ii) the mean RT was longest in the red cluster, and it increased from wave 1 to wave 3, while in the other clusters it decreased, (iii) there were no evident differences in the anxiety and depression inter-wave evolution between clusters, (iv) the red cluster had the highest percentage of participants with diabetes and/or taking anti-hypertensives. The inter-wave evolution of the main variables of the present study and how they differed among clusters are treated in more detail in the next section.

#### 3.4.2. Mobility and Cognitive Decline across Clusters

**Figure 8:**
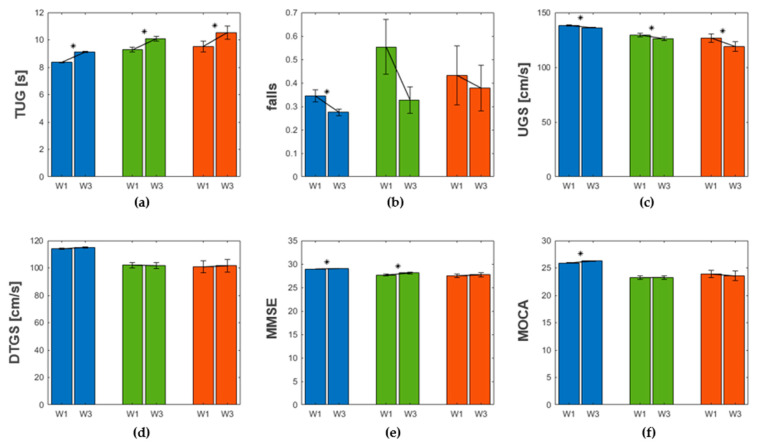
Evolution of TUG (**a**), number of falls reported (**b)**, UGS (**c**), DTGS (**d**), MMSE (**e**) and MOCA (**f**) from wave 1 to wave 3 specific for each cluster. The bars are coloured based on the corresponding cluster (blue, green and red, respectively). The height of the bars indicates the average value of the variable for a given cluster in a given wave. Stars indicate whether the difference of distributions of values of a given variable for a given cluster was statistically significant between waves.

Figure 8 shows in bar plots the inter-wave evolution of the main variables specific to each cluster. Error bars denote the standard error of the distribution of values of a given variable in a given wave for a given cluster. Stars indicate whether the difference of distributions of values of a given variable for a given cluster was statistically significant between waves. TUG significantly increased from wave 1 to wave 3 (Wilcoxon test p≤0.001) showing a mobility decline present in all clusters. The number of falls reported decreased in average from wave 1 to wave 3 for all clusters; however, only for the blue cluster was the difference between distributions of the two waves statistically significant (Wilcoxon test p=0.029). UGS significantly decreased from wave 1 to wave 3 for all clusters (Wilcoxon test p ≤ 0.002), agreeing with the mobility decline after 4 years already detected with TUG. DTGS remained the same for all clusters: no significant differences were found between the distribution in wave 1 and the distribution in wave 3 (Wilcoxon test p ≥ 0.279). MMSE increased from wave 1 to wave 3 in all clusters, but only for the blue and the green clusters was the difference between distributions of the two waves statistically significant (Wilcoxon test p ≤ 0.010). MOCA significantly increased after 4 years for the blue cluster (Wilcoxon test p<0.001), remained the same for the green cluster (Wilcoxon test p=0.999), and seemed to decrease for the red cluster, although with no statistical significance (Wilcoxon test p=0.579).

**Figure 9:**
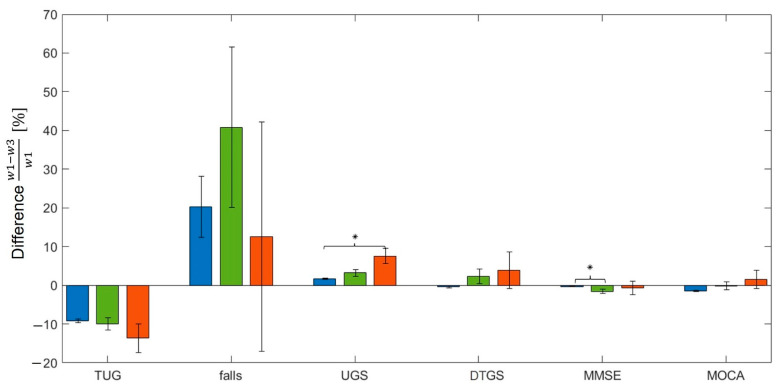
Comparison of the relative inter-waves change of TUG, falls, UGS, DTGS, MMSE and MOCA among clusters. Stars indicate whether the difference of distributions of inter-wave change values between clusters was statistically significant.

Figure 9 shows a comparison of the inter-wave change across clusters. Specifically, the height of the bars indicates the relative difference (relative inter-wave change) of a given variable between waves 1 and 3 for each cluster. Error bars indicate the standard error of the distribution of the relative inter-wave change values for each cluster. Stars indicate whether the difference of distributions of inter-wave change values between clusters was statistically significant. The inter-wave change for TUG was bigger for the red cluster, although the difference among clusters was not statistically significant (Mann–Whitney U test *p* ≥ 0.179 for all pairs). The inter-wave change for falls was bigger for the green cluster; however, there was no statistically significant difference between clusters (Mann–Whitney U test *p* ≥ 0.409 for all pairs). The inter-wave change for UGS was bigger for the red cluster, showing statistically significant difference to the inter-wave change in the blue cluster (Mann–Whitney U test p=0.004), while no significant differences were found for all other pairs (Mann–Whitney U test *p* ≥ 0.051). The inter-wave change for DTGS was bigger for the red cluster, but there was no statistically significant difference between clusters (Mann–Whitney U test *p* ≥ 0.188 for all pairs). The inter-wave change for MMSE was bigger for the green cluster, showing statistically significant difference with the inter-wave change in the blue cluster (Mann–Whitney U test p<0.001), while no significant differences were found for all other pairs (Mann–Whitney U test *p* ≥ 0.242). The inter-wave change for MOCA was negative for the blue cluster, indicating an improvement in the performance, null for the green cluster, indicating no change between waves, and positive for the red cluster, indicating a worsening of cognitive performance, although no significant differences were found between clusters (Mann–Whitney U test *p* ≥ 0.111 for all pairs).

**Figure 10:**
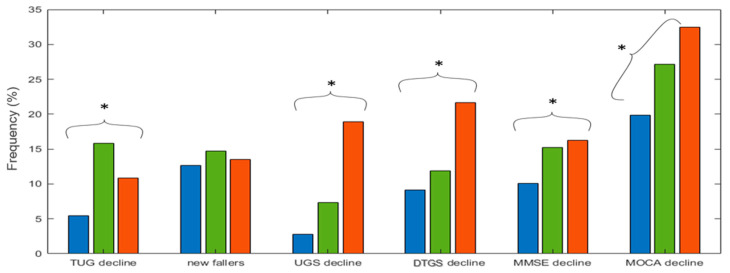
Percentage of TUG decline, new fallers, UGS decline, DTGS decline, MMSE decline and MOCA decline across clusters. The bars are coloured based on the corresponding cluster (blue, green and red, respectively). Stars indicate whether the condition of decline of a given participant statistically significantly depended on the classification in clusters, namely if the classification in clusters statistically significantly represented a difference in percentage of decline.

The last step of the comparison between clusters is presented in Figure 10. Here bars represent the frequency of decline of the main variables of the present study, as defined in Section 2.1.3 and Section 2.1.4, within each cluster. Stars indicate whether the condition of decline of a given participant statistically significantly depended on the classification in clusters, namely if the classification in clusters statistically significantly represented a difference in percentage of decline. Specifically, the presence of decline for TUG, UGS, DTUGS, MMSE and MOCA significantly depended on the classification in clusters ($\chi^{2}$-test p<0.013 for all variables but MMSE, for which the *p*-value was p=0.049), while the possibility of being a new faller did not significantly depended on the classification in clusters ($\chi^{2}$-test p=0.708). The blue cluster presented the lowest percentage of decline in all variables. Moreover, the green cluster presented a significantly higher percentage of TUG decline ($\chi^{2}=41.825$), while the red cluster presented a significantly higher percentage of decline in UGS ($\chi^{2}=44.971$), DTGS ($\chi^{2}=9.039$), MMSE ($\chi^{2}=6.051$) and MOCA ($\chi^{2}=8.644$). Therefore, based on the $\chi^{2}$-value, the mobility decline represented by a decline in TUG and UGS was highly dependent on the clusters’ classification.

## 4. Discussion

The SART has been widely utilized to investigate executive cognitive functions of healthy subjects and patients with neurodegenerative disorders [19,20]. Particularly, SART has been shown to be a valuable tool to explore the emergent awareness in patients of FTD, CBG and PSP, where direct frontal atrophy or breakdown of fronto-subcortical pathways contributed to the disruption to metacognitive awareness [19]. Moreover, the SART test has been used to investigate the sustained attention in robust, pre-frail, and frail older adults, employing frequency-spectral analysis techniques to associate the RT frequency bands with certain conditions [20]. It has been demonstrated that the fast variability component of sustained attention was strongly positively correlated with the risk of pre-frailty or frailty [17]. Considering the important role that SART plays in the medical research and clinical investigation of the assessment of executive functions, we expanded our previous results [7] in this study, employing new techniques for the longitudinal study of SART, and applying novel big data analysis algorithms to investigate potential correlations with other physiological systems.

### 4.1. Longitudinal Study of SART

#### 4.1.1. Longitudinal Multimodal Visualisation

In the present work, we employed a previously reported methodology for the multimodal visualisation of big repeated-measures data with continuous variable ordering, already introduced in a previous companion paper [7]. We applied the technique to the raw SART performance data, accompanied by global measures, such as MMSE score and TUG, and enriched the visualisation with a longitudinal approach, representing in the same panel the SART, MMSE, and TUG datasets at wave 1 and wave 3 using two different color-codes (Figure 2).

The advantages of the new visualisation are discussed elsewhere [7]. Briefly, this novel type of visualisation allows researchers and clinicians to appraise a large amount of information in ‘the blink of an eye’. The whole complex repeated-measures dataset (SART performances in this case) across different subjects, sorted by age, and across repeated measures, is represented in the same figure. Moreover, the additional presence of global parameters for diverse physiological systems could help clinicians to formulate relevant hypotheses that consider the general health status of the subject, analysed per se and in comparison with other subjects in the same age group.

The new element of the multimodal visualisation introduced in the present study allows cross-sectional and longitudinal comparisons in the same figure. Using the thresholding variable, as previously introduced [7], and the double colour-code, we can analyse the dynamic change of ‘big spots’ density from wave 1 to wave 3 across different age groups. For example, considering the merged cohort of waves 1 and 3, we note that 68% of participants who had at least one bad performance at wave 1, did not have any bad performances at wave 3, so they improved their SART performances, while the remaining 32% maintained a number of BP ≥ 1 at wave 3, constituting the 26% of the participants with bad performances. On the other hand, 74% of this latter subgroup showed a worsening of their SART performance from wave 1 to wave 3, going from a value BP = 0 to BP > 0. We can also notice that the total number of mistakes increased from wave 1 to wave 3. Thus, having in the same figure the multimodal SART visualisation at wave 1 (a) and at wave 3 (b), and the subset of SART bad performances for waves 1 and 3 differentiated by colour in the same graph (c), could help to understand how the SART performances of participants in the merged cohort evolve after 4 years. We were, therefore, interested in understanding which physiological factors at wave 1 could have predicted a worsening of SART performance at wave 3.

#### 4.1.2. Predictive Model for SART Performance after 4 Years

As we mentioned in the previous section, the distribution of ‘big spots’ changed from wave 1 to wave 3. Not only the number of participants with bad performances increased, but also the number of bad performances per participant increased at wave 3 and, above all, a consistent portion of this subgroup was constituted by participants who did not have bad performances at wave 1. The results of our statistical study on the inter-wave change of BP are widely presented in Results Section 3.2. Our findings showed a generally significant worsening of SART performances after 4 years. Wilcoxon rank sum test suggested a significance difference between the distributions of BP at waves 1 and 3 (*p* < 0.001), and a low Spearman’s coefficient (ρ=0.210 at the significance level α=0.01) demonstrated the absence of significant trends between $BP_{1}$ and $BP_{3}$. Moreover, recent studies [7,25,27,31] have shown interactions between different physiological systems and, particularly, correlations have been found between cognitive and mobility decline. Therefore, motivated to inspect if predictors of this worsening could be found in other physiological systems, we employed BLR models, having as output the dichotomised variable that represented the presence of bad performances at wave 3 (see Section 2.4.1 and Section 3.3).

Based on the same structure of the BLR models employed in [7], we considered BLR models using an increasing number of independent variables (see Section 2.4.1) besides the main predictor. As main predictor we used the number of bad performances at wave 1, after testing the variable ‘bad performances’ at wave 1 and wave 3 for potential trends (Spearman’s rank correlation coefficient ρ=0.210 at the significance level α=0.01), the total number of SART mistakes at wave 1 and the number of mistakes in good performances at wave 1. The BLR models considered the main predictors separately, namely the three main predictors did not figure together in the same model: each time a BLR model had a main predictor and an increasing number of covariates as independent variables. The reason behind this choice was that we were interested in understanding which variable had good predictive power for the outcome and could be used independently from the other predictors.

Our findings showed that in all combinations of covariates the $BP_{1}$ had the highest OR with no overlapping 95% confidence intervals, demonstrating, indeed, the highest predictive power to detect the presence of bad performances at wave 3. Other significant predictors were SD RT, age, and level of anxiety, while the third or higher level of education was significantly protective against $BP_{3}>0$. We note that the significance of these predictors was always very high, having a *p*-value equal or lower than 0.005, and showing, then, the high reliability of our findings.

Previous findings [7] showed the importance of the variable ‘bad performances’ to characterise cognitive performance and to predict a mobility decline after 4 years. Here, we demonstrated the importance of this variable in the prediction of a worsening of the SART performance, and we believed that this could have further clinical implications. We used a combination of $BP_{1}$ and $BP_{3}$ in a longitudinal study to detect a subgroup of subjects who would show a decline in multiple physiological systems. The concept of multiple physiological dysregulations underlines frailty, a complex geriatric syndrome, which is manifested in older adults as a general decline in different organs [22]. Frailty could be seen in its physical dimension, where certain symptoms like muscle weakness, slow gait speed, and weight loss are the natural markers, and the cognitive dimension, where the focus is the assessment of cognitive status [70]. Therefore, recent findings proposed a multidimensional approach, investigating the role of different neuropsychological domains to individuate frailty and pre-frailty [71]. Particularly, significant associations between frailty and action monitoring, depression and disinhibition, and impaired awareness for instrumental activities disabilities have been found [71]. In the framework of this multidimensional approach for investigation of frailty, the present research work aims to detect, through an automatic algorithm based on the evolution of SART performance in a 4 years-time frame, specific groups of people who show decline at different physiological levels.

### 4.2. Fuzzy Clusters and the Three Degrees of Physiological Dysregulation

The natural tool of investigation was the application of clustering techniques. Clustering methods are nowadays frequently used in biological and medical research [39,42] aiming to automatically individuate subsets of subjects with similarities, which could represent particular physiological conditions, or subsets of values in physiological parameters, which could represent biological markers. Our aim was to develop a clustering technique that would allow to high specificity in the dataset analysed, individuating a small group of participants with the highest risk of decline and potentially in need of closer medical attention.

Recent studies have demonstrated that hard clustering algorithms were not well suited to the analysis of biomedical data in most situations, since the subdivision in a certain dataset between a subset of participants with a potential disorder and a subset of participants who were potentially healthy was not very clear, for a variety of reasons [42]. Therefore, the fuzzy clustering with its probabilistic approach seemed to reproduce more faithfully what happens in reality. In hard clustering, the algorithm partitions the set well when the clusters are dense and well separated, namely when the elements of each cluster are close enough to the centroid and sufficiently far from other cluster centroids. Differently, in a fuzzy clustering algorithm such as FCM, the centroid of each cluster is attracted towards outliers instead of the center of the cluster. The latter is, therefore, more suitable when the partition is not clear for the intrinsic nature of the data.

We employed the FCM algorithm to classify the merged cohort (waves 1 and 3) into 3 clusters based on the evolution of the variable ‘bad performances’ from wave 1 to wave 3. We were able to individuate 3 different degrees of physiological dysregulation in different physiological systems, represented by the 3 clusters created: $C_{1},\;C_{2}$, $C_{3}$, blue, green and red, respectively. The blue cluster, the biggest (94% of the entire cohort), comprehended participants with a low number of bad performances and total mistakes at both waves, youngest mean age, with the fastest RT and lowest SD RT. We note that the SART-related variables indicate an improvement of SART performance from wave 1 to wave 3, although very small. Moreover, compared to the other clusters at both waves, the blue cluster comprehended participants with shortest TUG, highest UGS and DTGS, lowest number of falls reported, highest MMSE and MOCA scores, lowest percentage of participants with primary or one level of education and highest percentage of participants with third/higher level of education, lowest percentage of participants with diabetes or on anti-hypertensives, but high percentage of participants with a drinking problem (Table 5). The green cluster (5% of the entire cohort) comprehended participants with a high number of bad performances at wave 1 but with a lower number of bad performances at wave 3. Consequently, even the number of total mistakes substantially decreased from wave 1 to wave 3 and the mean RT and SD RT were lower in wave 3 compared to wave 1, indicating a considerable improvement of the SART performance. Moreover, the green cluster had the highest percentage of female participants, the lowest percentage of smokers and the lowest percentage of participants with a drinking problem at wave 1. However, compared to the other clusters, the participants in the green cluster had the lowest MOCA score, the highest level of anxiety and depression (wave 1), the highest percentage of participants with primary/no education and low level of physical activity, especially at wave 3, which could explain the biggest drop of the number of falls reported compared to the other clusters (Table 5). Furthermore, the green cluster was the only one where the DTGS, averaged across participants, decreased from wave 1 to wave 3. Finally, the red cluster (only 1% of the entire cohort), comprehended participants with none or a low number of bad performances at wave 1, but with a very high number of bad performances at wave 3. The number of total mistakes for this cluster increased by 682%, from 17.7 at wave 1 to 120.7 at wave 3; besides, this was the only cluster where the mean RT and the SD RT increased at wave 3 compared to wave 1, indicating a general steep worsening of the SART performance. Moreover, compared to the other clusters at both waves, the participants of the red cluster had the longest TUG, the lowest UGS and DTGS, the lowest MMSE score, and the biggest increase of depression level, from the lowest value at wave 1 (5.0) to the highest value at wave 3 (3.6); this cluster was the only one where the MOCA scores decreased from wave 1 to wave 3, and had the highest percentage of participants with secondary level of education, diabetes, anti-hypertensives, and the highest percentage of past or current smokers. Moreover, this cluster had the biggest drop in the percentage of participants with high level of physical activity, from the highest value at wave 1 (37.8%) compared to the other clusters to the lowest at wave 3 (19.4%). The only healthy signs for participants in the red cluster were the lowest level of anxiety and smallest number of participants with a drinking problem, but even if in this case the percentage of the dummy group was high (Table 5).

Summarising, we could consider the blue cluster as constituted by the healthier majority of the sample, according to diverse physiological parameters, and the green cluster as constituted by a small portion of participants whose SART performance markedly improved, especially taking into account that this cluster showed the lowest MOCA score at both waves, the highest level of anxiety and the highest percentage of participants with none/primary level education. Moreover, the participants in the green cluster also showed an improvement regarding the level of depression and smoking status, having the highest percentage of participants (6%) transitioning from the status of current to past smoker after 4 years. On the other hand, the red cluster revealed a selected group of participants who showed a dramatic worsening of the SART performance after 4 years, especially considering the low level of anxiety, one of the significant predictors for the presence of bad performances at wave 3 according to the BLR models (see Section 3.3). Furthermore, the participants in the red cluster showed a multiple dysregulation, a general unhealthy status in all physiological systems, especially at wave 3, suggesting certain common features to identify subjects who might need to be kept under medical observation.

#### High Specificity for a Selective Group of High-Risk Participants

As mentioned in the previous section, the FCM algorithm allowed detection of a very restricted group of participants who showed dysregulation in multiple physiological systems. This is not only a cross-sectional observation, but also a longitudinal consideration. The comparison among clusters for the main variables is presented at three different levels of inspection in Section 3.4.2.

Figure 8 shows the main variables and their evolution from wave 1 to wave 3 for all clusters. All showed a homogeneous trend in mobility decline, represented by an increase in TUG and decrease in UGS, and a general learning effect considering the cognitive variables, consistent with other findings [72,73]. Moreover, the decrease in the number of falls reported, manifested in all clusters, could be seen in relation to the general decrease in the percentage of participants with higher levels of physical activity. We note that the increase of MMSE score, due to a learning effect, is not significant for the red cluster; besides, the increase of MOCA score is significant only for the blue cluster, while the red even showed even a decrease in this score.

Figure 9 shows a comparison of the relative differences between wave 1 and wave 3 of the main variables among clusters. The biggest difference between waves was represented by the red cluster, with the exception of number of falls reported, which reported a high SD for all clusters, and the MMSE score. Moreover, the red cluster was the only one with a positive relative difference between wave 1 and wave 3, indicating a decrease of MOCA score after 4 years. Due to a general large SD in the relative inter-wave change for most of the variables, a significant difference in inter-wave change among cluster was found only in two cases: between the blue and the red cluster for UGS decline, and between the blue and the green cluster for the improvement in MMSE score. The latter case indicated that the biggest improvement concerning the MMSE score was found in the green cluster, further suggesting that the green cluster was mainly constituted by participants who showed a substantial improvement in cognitive status.

Finally, Figure 10 shows a comparison of the percentage of participants with decline for the main variables, as defined in Section 2.1.3 and Section 2.1.4, between clusters. The red cluster presented the highest percentage of participants with mobility and cognitive decline, with the exception of TUG decline and new fallers, where the highest percentage of decline was found in the green cluster. Moreover, for all main variables, with the exception of new fallers, the classification in clusters statistically significantly represented a difference in percentage of decline. We mention that the statistical tests on the inter-wave change for the main variables across different clusters (Figure 8 and Figure 9) and on the percentage of decline across clusters (Figure 10) detected a very high effect size (Wilcoxon test p≤0.029 for significant differences between waves, Mann–Whitney U test p≤0.004 for significant differences of the inter-waves change between clusters, $\chi^{2}$-test p<0.013 for significant differences of the portion of decline between clusters, except for MMSE, which had a borderline p=0.049, and $\chi^{2}\geq 6.051$), indicating the high robustness of our findings on the population study. Therefore, the employed ‘physiologic clustering’ (i) individuated different degrees of physiological dysregulation in diverse physiological systems, and (ii) individuated in a generally healthy cohort a selected group of participants who presented a mobility and cognitive decline after 4 years. We note that a potential mobility decline would be easily detectable, while a cognitive decline is in general hard to detect [7]. Indeed, in population-based longitudinal studies such as TILDA, cognitive decline is difficult to detect, but a loss of the expected learning effect may signify clinically significant cognitive impairment despite no/mildly statistically significant differences in cognitive scores. The applied technique provided a high degree of specificity and in practice could potentially help clinicians select a small number of individuals to keep under medical observation.

### 4.3. Strengths and Limitations of the Study

One of the main strengths of our study is the possibility of working with a large dataset and comprehensive health assessment: TILDA is one of the most detailed population-based longitudinal studies of ageing, and the comprehensive measures and tests taken at different waves constitute the main strength for longitudinal analyses involving various physiological systems. In particular, a complex repeated measures dataset, like SART, allows deep investigation for a large sample of individuals. Moreover, the predominant longitudinal aspect of the TILDA study allows investigations over time, providing a further dimension to cross-sectional studies, and allowing the investigation of the dynamic evolution of various physiological parameters. Furthermore, the FCM algorithm applied to the new variable ‘bad performances’ at waves 1 and 3 allowed detection in a large generally healthy cohort of a very specific group of participants that might require closer monitoring, because after four years they presented marked signs of dysregulation across multiple physiological systems.

Our study also has potential limitations. For example, in this study we did not investigate sensorium, nor did we make a comprehensive neuropsychological assessment, including of autonomy in daily life, awareness of possible deficits, or an exploration of individual cognitive domains, although previous studies had shown correlations between sustained attention and preferred retinal locus, a fundamental compensative mechanism in patients with foveal vision loss [74]. We focused on variables which are mainly representative of the mobile and cognitive systems. This is a first step in longitudinal investigation of the complex raw information contained in the SART dataset, and its possible correlations with other physiological systems. Future studies will consider the entire spectrum of cognitive functions for the different clusters of participants and the analysis of their brain magnetic resonance imaging (MRI), which has been shown to contain useful information and clear biomarkers of accelerated brain ageing and neurodegenerative disorders [18,75]. Indeed, a GO-NoGO fMRI paradigm investigated metacognitive-executive functions in neurocognitive disorders and in neuropsychiatric diseases, e.g., in Parkinson’s disease, in which the loss of dopaminergic neurons impacts on the functioning of ACC, which has a central role in detecting the processing of conflict, intention, and response initiation/inhibition [17,18].

Moreover, the green and, especially, the red clusters had a very low number of participants, which affected the statistical analysis and made for difficulty of interpretation in comparison with the large blue cluster. Of the entire cohort, 94% fell into the blue cluster; this represented the vast majority of healthier participants, this being this a population study conducted on relatively healthy and high-functioning older adults. The participants in the blue cluster basically did not have worrying SART performances at both waves, nor did they show signs of decline in the other physiological systems. In contrast, the remaining participants represented the small portion of adults that improved their SART performances and their life in general (green cluster), and a very select group of people that not only dramatically worsened in the SART, but also manifested multiple organic dysregulation (red cluster). We note that all the statistical tests in the present study always referred to the size of each cluster. In fact, some results did not reach statistical significance. However, we believe that this is a small price worth paying for having obtained a selected group of participants who showed mobility and, most importantly, cognitive decline after 4 years. This is usually hard to detect [76], especially in relatively high-functioning community-dwelling adults with good cognitive and physical health [77]. In TILDA, we succeeded in identifying a very small group of participants at high risk of physical and cognitive decline after four years. Translating this to clinical practice, our findings mean that our methodology could be replicated to allow clinicians to identify highly specific patients who may require closer medical follow up and interventions to prevent accelerated loss of functionality and premature loss of independence.

## 5. Conclusions

In conclusion, the present work expanded the multimodal visualisation previously introduced in [7] with a longitudinal approach, allowing (i) rapid visual inspection of a large amount of data, the complex raw SART data in this case, and to identify poor SART performances, (ii) inspection of the dataset together with different health variables of clinical interest, and (iii) observation of evolution across waves in the same graph. This representation would allow researchers and clinicians to compare the participants’ performances between each other and across time in order to generate hypotheses. Moreover, the study offered a longitudinal inspection of the SART dataset, investigating main predictors for the presence of BP at wave 3, and individuating as such $BP_{1}$, age and level of anxiety. Furthermore, applying the fuzzy clusters algorithm to the evolution of the variable bad performances from wave 1 to wave 3, we were able (i) to automatically organise the participants into three different groups based on their SART performances at waves 1 and 3, (ii) individuate three different degrees of physiological dysregulation, represented by healthy participants (blue cluster), participants whose cognitive status was not the highest compared to the rest of the dataset but who showed the biggest improvement (green cluster), and participants whose mobility and cognitive conditions steeply deteriorated after 4 years (red cluster); and (iii) to identify a very specific group of participants that might require closer monitoring, because after four years they presented marked signs of dysregulation across multiple physiological systems. The identification of such a group of participants in a cohort of relatively high-functioning community-dwelling adults with good cognitive and physical health is the very first step in the detection of frailty, in the framework of a multidimensional approach based on metacognitive-executive functions [71].

## Figures and Tables

**Table 1:** Descriptive statistics for the whole set of variables considered in this study for the merged dataset (N=3468) at wave 1 and wave 3. The first part of the table gives minimum, maximum values, and mean and SD for each continuous variable. The second part shows ordinal or nominal variables and their frequency in percentage.

Continuous Variable	Wave 1: Mean (SD); Range	Wave 3: Mean (SD); Range
SART bad performances	0.2 (0.8);	0.4 (1.8);
	0–15	0–23
SART: Total mistakes	9.6 (10.6);	11.0 (16.0);
	0–92	0–184
SART: Mistakes in good performances	8.8 (8.8);	8.9 (8.6);
	0–60	0–51
SART: Mean RT (ms)	381.4 (94.2);	348.4 (84.8);
	168.9–836.5	156.0–842.0
SART: SD RT (ms)	69.7 (40.1);	71.0 (43.3);
	12.7–364.2	0.0–347.4
TUG (s)	8.6 (1.7);	9.2 (2.1);
	4.8–28.5	5.1–27.6
UGS (cm/s)	137.6 (19.0);	135.4 (20.2);
	43.1–207.5	47.5–207.5
DTGS (cm/s)	113.2 (25.6);	114.4 (25.6);
	28.4–203.4	26.2–203.3
Falls	0.4 (1.4);	0.1 (4.2);
	0–50	0–15
MMSE	28.9 (1.6);	29.0 (1.4);
	0–30	15–30
MOCA	25.7 (2.9);	26.9 (3.0);
	7–30	7–30
Age (years)	61.0 (7.8);	65.3 (7.7);
	50–89	53–94
Anxiety	5.4 (3.5);	8.0 (2.6);
	0–20	6–23
Depression	5.3 (6.6);	3.0 (3.6);
	0–48	0–24
**Ordinal/Nominal Variable**	**Cohort 1 (Wave 1) ** **Frequency (%)**	**Cohort 2 (Merged Wave 1–3) ** **Frequency (%)**
Female	54.2	54.2
Education level		
- primary/none	17.5	17.4
- secondary	41.9	39.9
- third/higher	40.6	42.7
Anti-hypertensives	30.4	39.3
Diabetes	5.4	7.0
Smoker		
- never	47.3	47.1
- past	39.5	43.4
- current	13.2	9.5
Drinking problem	13.8 (7.4 *)	12.4 (10.5 *)
IPAQ		
- low	26.2	33.0
- medium	36.6	36.4
- high	37.3	25.4

* Dummy group of participants who answered “Don’t know” to the question “Do you have a drinking problem?”.

**Table 2:** Summary of distribution density for the individual trial mistakes at wave 1 and wave 3.

Number of Mistakes within a Trial	Wave 1		Wave 3		Change between Wave 1 and Wave 3 [%]	
	N. Participants	Total	N. Participants	Total	N. Participants	Total
0	3444	60,346	3433	59,403	−0.3%	−1.6%
1	2736	9035	2764	9396	+1.0%	+4.0%
2	2522	7925	2599	7844	+3.1%	−1.0%
3	925	1854	964	1877	+4.2%	+1.2%
4	268	429	323	491	+20.5%	+14.5%
5	79	100	98	135	+24.1%	+35.0%
6	22	32	42	58	+90.9%	+81.3%
7	19	24	44	66	+131.6%	+175.0%
8	4	4	66	494	+1550%	+12,250%
9	13	15	0	0	−100%	−100%

**Table 3:** Comparison of the OR and corresponding 95% C.I. of bad performances, total mistakes, and mistakes in good performances for the prediction of bad performances w3 in the binary logistic regression models.

Bad Performances w3									
	Bad Performances			Total Mistakes			Mistakes in Good Performances		
	OR	95% C.I.	*p*	OR	95% C.I.	*p*	OR	95% C.I.	*p*
Model 1	1.673	1.476–1.896	<0.001	1.065	1.056–1.074	<0.001	1.077	1.067–1.088	<0.001
Model 2	1.364	1.216–1.530	<0.001	1.054	1.043–1.065	<0.001	1.063	1.049–1.077	<0.001
Model 3	1.301	1.159–1.461	<0.001	1.045	1.033–1.057	<0.001	1.051	1.036–1.066	<0.001
Model 4	1.326	1.167–1.506	<0.001	1.044	1.032–1.057	<0.001	1.049	1.033–1.065	<0.001

Models for each main predictor, i.e., bad performances, total mistakes, or mistakes in good performances: model 1, with just the main predictor; model 2, adjusted with mean RT and SD RT; model 3, which is model 2 with the addition of age, sex, and education level; model 4, the fully adjusted regression model, considering also the other covariates mentioned in Section 2.1.5 (anxiety, depression, hyper-tensives, diabetes, smoking, alcohol, UGS at baseline (wave 1), and IPAQ). The odds ratio (OR) and corresponding 95% confidence interval (C.I.) give a measure of the influence of the predictor on the outcome; the *p*-value expresses the statistical significance of the predictor in the model.

**Table 4:** Results for the fully adjusted per covariates binary logistic regression model 4 considering bad performances as potential predictor of having SART bad performances at wave 3.

Bad Performances w3			
Independent Variable	OR	95% C.I.	*p*-Value
Bad performances w1	1.326	1.167–1.506	<0.001
SART mean RT	1.000	0.999–1.001	0.931
SART SD RT	1.009	1.006–1.011	<0.001
Age	1.070	1.052–1.088	<0.001
Females	0.939	0.727–1.212	0.629
Education level			
- primary/none	[ref]		
- secondary	0.920	0.679–1.245	0.588
- third/higher	0.580	0.417–0.805	0.001
Anxiety	1.057	1.017–1.099	0.005
Depression	1.001	0.981–1.022	0.926
Anti-hypertensives	0.888	0.681–1.158	0.381
Diabetes	1.399	0.877–2.233	0.158
Smoker			
- never	[ref]		
- past	1.021	0.786–1.325	0.877
- current	1.155	0.784–1.702	0.466
Drinking problem			
- “No”	[ref]		
- “Don’t know”	1.263	0.515–3.100	0.610
- “Yes”	0.740	0.498–1.100	0.136
UGS at baseline	0.994	0.987–1.001	0.081
IPAQ			
- low	[ref]		
- medium	1.173	0.868–1.586	0.300
- high	1.046	0.763–1.435	0.778

**Table 5:** Descriptive statistics for the whole set of variables considered in this study at wave 1 and wave 3 for each cluster. The first part of the table gives minimum, maximum values, and mean and SD for each continuous variable. The second part shows ordinal or nominal variables and their frequency in percentages.

Continuous Variable	Cluster Blue (*N* = 3254)		Cluster Green (*N* = 177)		Cluster Red (*N* = 37)	
	Wave 1: Mean (SD); Range	Wave 3: Mean (SD); Range	Wave 1: Mean (SD); Range	Wave 3: Mean (SD); Range	Wave 1: Mean (SD); Range	Wave 3: Mean (SD); Range
Age (years)	60.6 (7.6);	65.0 (7.6);	65.8 (8.5);	70.2 (8.5);	68.7 (7.5);	73.0 (7.6);
	50–89	53–94	50–85	54–89	50–86	54–90
SART bad performances	0.1 (0.2);	0.1 (0.4);	2.4 (2.2);	1.8 (2.3);	0.7 (2.0);	15.5 (4.6);
	0–1	0–4	0–15	0–9	0–11	9–23
SART: Total mistakes	8.2 (8.1);	8.7 (8.9);	33.5 (16.6);	11.0 (16.0);	17.7 (17.7);	120.7 (36.3);
	0–64	0–55	2–92	0–184	0–74	64–184
SART: Mistakes in good performances	8.8 (8.8);	8.2 (8.0);	22.7 (12.0);	8.9 (8.6);	14.7 (12.3);	8.4 (8.6);
	0–60	0–51	0–51	0–51	0–46	0–32
SART: Mean RT (ms)	376.7 (91.3);	343.1 (81.1);	459.6 (108.0);	348.4 (84.8);	422.6 (101.7);	438.1 (95.6);
	168.9–794.7	156.0–842.0	232.6–836.5	156.0–842.0	238.5–625.9	246.9–668.4
SART: SD RT (ms)	66.6 (37.1);	63.3 (41.4);	121.1 (50.7);	71.0 (43.3);	99.5 (59.6);	110.3 (60.2);
	12.7–364.2	9.7–347.4	25.3–302.0	0.0–347.4	34.0–290.7	0.0–256.5
TUG (s)	8.4 (1.6);	9.1 (2.0);	9.3 (2.3);	10.1 (2.5);	9.5 (2.3);	10.5 (2.8);
	4.8–28.5	5.1–27.6	5.6–24.3	6.2–18.4	6.3–17.6	6.7–18.1
UGS (cm/s)	138.2 (18.6);	136.1 (19.9);	129.5 (22.1);	126.1 (21.7);	126.7 (23.4);	119.0 (25.3)
	43.1–207.5	47.5–207.5	46.0–181.3	63.8–177.6	64.2–164.3	67.4–161.6
DTGS (cm/s)	113.9 (25.4);	115.2 (25.3);	102.1 (25.5);	101.7 (28.2);	100.8 (26.7);	101.6 (24.0);
	28.4–203.4	26.2–203.3	34.4–167.6	26.2–179.3	39.9–140.5	51.2–146.5
Falls	0.3 (1.4);	0.3 (0.8);	0.6 (1.6);	0.3 (0.8);	0.4 (0.8);	0.4 (0.6);
	0–50	0–15	0–12	0–4	0–3	0–2
MMSE	29.0 (1.5);	29.0 (1.3);	27.7 (2.6);	28.1 (2.3);	27.5 (2.5);	27.7 (3.1);
	0–30	19–30	19–30	18–30	20–30	15–30
MOCA	25.9 (2.7);	26.3 (2.8);	23.2 (4.0);	23.3 (4.2);	23.9 (4.2)	23.5 (5.2);
	13–30	11–30	10–30	7–30	7–30	9–30
Anxiety	5.4 (3.5);	8.0 (2.6);	5.7 (3.5);	8.3 (2.7);	5.2 (3.9);	7.6 (1.7);
	0–20	6–23	0–19	6–21	0–15	6–11
Depression	5.2 (6.6);	3.0 (3.6);	5.9 (6.6);	3.3 (3.6);	5.0 (7.2);	3.6 (4.0);
	0–48	0–24	0–31	0–20	0–33	0–15
**Ordinal/Nominal Variable**	**Wave 1 ** **Frequency** **(%)**	**Wave 3 ** **Frequency** **(%)**	**Wave 1 ** **Frequency** **(%)**	**Wave 3 ** **Frequency** **(%)**	**Wave 1 ** **Frequency** **(%)**	**Wave 3 ** **Frequency ** **(%)**
Female	54.1	54.1	58.8	58.8	40.5	40.5
Education level						
- primary/none	16.2	16.1	39.0	39.5	27.0	27.0
- secondary	42.1	40.0	37.3	36.7	48.6	45.9
- third/higher	41.7	43.9	23.7	23.7	24.3	27.0
Anti-hypertensives	29.8	38.8	35.0	42.4	56.8	70.3
Diabetes	5.3	6.8	6.2	7.9	13.5	16.2
Smoker						
- never	47.1	46.9	51.4	51.4	43.2	40.5
- past	39.7	43.5	35.6	41.2	43.2	45.9
- current	13.2	9.6	13.0	7.3	13.5	13.5
Drinking problem	14.0 (7.2 *)	12.6 (10.3 *)	10.2 (10.7 *)	9.6 (12.4 *)	13.5 (5.4 *)	2.7 (16.2 *)
IPAQ						
- low	25.9	34.4	31.4	41.4	27.0	38.9
- medium	36.5	38.5	37.1	36.4	35.1	41.7
- high	37.6	27.1	31.4	22.2	37.8	19.4

* Dummy group of participants who answered “Don’t know” to the question “Do you have a drinking problem?”.

## Data Availability

The datasets generated during and/or analysed during the current study are not publicly available due to data protection regulations, but are accessible at TILDA on reasonable request. The procedures to gain access to TILDA data are specified at https://tilda.tcd.ie/data/accessing-data/ (accessed on 6 September 2021).

## Appendix A

The FCM algorithm partitions a collection of N elements $S=\left\{{\underline{x}}_{i,i=1,\ldots ,N}\right\}$, defined in a space where it is possible to state a metric *d*, into *c* fuzzy groups, such that c≤N [39,61,78].

Similarly to hard clustering [4], the FCM algorithm follows an iterative procedure with random initialization of cluster centroids ${\underline{q}}_{i,i=1,\ldots ,c}$. The centroids are iteratively updated as the remaining elements of *S* are assigned to the clusters, where the membership matrix $U=\left[u_{ij}\right]\in M^{c\times N}$, having as many rows as there are clusters, and as many columns as there are elements of *S*, and which indicates the degree of belongingness of each element to each cluster, is such that:

1. $0\leq u_{ij}\leq 1\;\forall i\in 1,\ldots ,c,\forall j\in 1,\ldots ,N$
2. $\overset{c}{\underset{i=1}{\sum }}u_{ij}=1\;,\;\overset{N}{\underset{j=1}{\sum }}u_{ij}>0$
3. the objective function of FCM $J_{FCM}=\overset{c}{\underset{i=1}{\sum }}\overset{N}{\underset{j=1}{\sum }}u_{ij}{}^{m}\;d{\left({\underline{x}}_{j,\;}{\underline{q}}_{i}\right)}^{2}\;$ is minimised.

where *m* is the *fuzziness* parameter: the higher it is, the more likely are the elements to belong to more clusters [39,61,78]. The fuzzy partitioning is carried out through an iterative optimization of the objective function, updating the membership $u_{ij}$ and the centroids ${\underline{q}}_{i}$ according to the following equations:

(A1) $$u_{ij}=\frac{1}{\sum_{k=1}^{c}{\left[\frac{d\left({\underline{x}}_{j,\;}{\underline{q}}_{i}\right)}{d\left({\underline{x}}_{j,\;}{\underline{q}}_{k}\right)}\right]}^{\;\;2/\left(m-1\right)}}$$

(A2) $$q_{i}=\frac{\sum_{j=1}^{N}u_{ij}{}^{m}x_{j}}{\sum_{j=1}^{N}u_{ij}{}^{m}}$$

We applied the FCM algorithm to classify the set of participants into three clusters, using the function *cmean* in MATLAB (R2020b, The MathWorks, Inc., Natick, MA, USA). In our case:

- the set of elements to partition is the merged cohort (wave 1 and 3) of participants
- the metric *d* has two components: the variable bad performances at wave 1 and the same variable at wave 3
- c=3
- m=2 (as default in MATLAB)
